# Interaction in Li@Fullerenes and Li^+^@Fullerenes: First Principle Insights to Li-Based Endohedral Fullerenes

**DOI:** 10.3390/nano9040630

**Published:** 2019-04-18

**Authors:** Hongcun Bai, Hongfeng Gao, Wei Feng, Yaping Zhao, Yuhua Wu

**Affiliations:** State Key Laboratory of High-efficiency Utilization of Coal and Green Chemical Engineering, College of Chemistry and Chemical Engineering, Ningxia University, Yinchuan 750021, Ningxia, China; hongfenggao@foxmail.com (H.G.); weifeng199411@foxmail.com (W.F.); lucky19950318@foxmail.com (Y.Z.); yuhua236@foxmail.com (Y.W.)

**Keywords:** lithium ion, endohedral fullerenes, reduced density gradient, energy decomposition analysis, DFT

## Abstract

This work reveals first principle results of the endohedral fullerenes made from neutral or charged single atomic lithium (Li or Li^+^) encapsulated in fullerenes with various cage sizes. According to the calculated binding energies, it is found that the encapsulation of a single lithium atom is energetically more favorable than that of lithium cation. Lithium, in both atomic and cationic forms, exhibits a clear tendency to depart from the center in large cages. Interaction effects dominate the whole encapsulation process of lithium to carbon cages. Further, the nature of the interaction between Li (or Li^+^) and carbon cages is discussed based on reduced density gradient, energy decomposition analysis, and charge transfer.

## 1. Introduction

With the development of micro- and nanotechnology, electronics in 21st century have become smaller and smaller. As a result, the power supply places a very high requirement on micro and nano electronics [1]. The lithium batteries including insertion compounds were first proposed by Michel Armand in 1970s [2]. Nowadays, lithium batteries have become standard in micro power supply due to their many advantages [3,4]. For instance, lithium batteries have high energy density, and they weigh half as much as nickel-cadmium or nickel-hydrogen batteries of the same capacity. The volume of lithium batteries is 20–30% that of nickel-cadmium batteries, and 35–50% that of nickel-hydrogen batteries. The working voltage of a single lithium-ion battery is high voltage (3.7 V average for Li and Li^+^), which is equivalent to three series nickel-cadmium or nickel-hydrogen batteries. Besides, the charging and discharging cycles of lithium-ion batteries can exceed 500 times, and those made with lithium iron phosphate can reach 2000 cycles under normal conditions. They also show fast charging: newly developed lithium-iron-phosphate batteries can be charged in 35 min. Additionally, lithium batteries do not contain harmful metals such as cadmium, lead or mercury.

On the other hand, lithium metal shows highly reactive chemical characteristics. This requires high environmental requirements for the processing, preservation and use of lithium [5,6]. Moreover, safety performance requirements are getting higher and higher for lithium batteries. It is increasingly important for batteries to show improved control sensitivity, more sensitive control parameters, and joint control of multiple parameters. In addition, the underlying technology of lithium-ion batteries used in such devices as computers and mobile phones still needs to be improved. Thus, more secure materials and structures need to be considered.

In order to develop lithium batteries with better performance and safety ratings, various materials have been studied [7,8,9,10]. Fullerenes [11], an important class of low-dimensional carbon-based nanomaterial, have attracted much attention due to their excellent physical and chemical properties [12,13,14,15]. According to the concept of qualitative design in composite materials, advanced materials with high measures of performance and safety will be obtained if the excellent properties of lithium and fullerenes are combined. It has been revealed that there is porous space available within the carbon nanocages; thus, fullerenes could be treated as nanoscaled containers to confine some species inside [16,17,18,19,20]. To date, various species have been encapsulated in fullerenes. 

Small metal clusters, especially the neutral or charged single atom, are unstable and highly reactive in free space. However, they could be stabilized well in the confined environment inside fullerenes [19,20,21,22]. As for Li-based species, Aoyagi et al. have successfully prepared a unique two-dimensional crystal structure of [Li^+^@C_60_] salt [23]. This unique Li-based endohedral fullerene may exhibit interesting solid-state properties. For instance, the containing structure of the carbon cage could protect atomic lithium from external agents. As a result, Li-based endohedral fullerenes with novel electronic properties may be good candidates for modeling the design of nano-scale lithium batteries.

Besides trial-and-error experiments, modern first-principle calculations also pay close attention to Li-based endohedral fullerenes [24,25,26,27,28]. The electronic properties of neutral and charged Li@C_60_ have been studied using density functional theory (DFT) calculations, finding that the C_60_ cage can accommodate three excess electrons [24]. Super-alkalis FLi_2_, OLi_3_ and NLi_4_ are found to be stable in C_60_ according to DFT results by Srivastava and the colleagues [25]. The impacts of Li on structural, dynamical, doping, optical and thermodynamic properties of the C_60_ fullerene have also been investigated using the framework of DFT [26,27,28]. 

Until now, however, a first-principle view remains unavailable for interactions between single-atom lithium (neutral or charged: Li or Li^+^) and fullerenes, and that view is particularly important in order to explore the behavior of the materials interface for batteries. This raises two questions. First, what are the energy and stability ratings of Li-based endohedral fullerenes with various sizes? Second, what is the nature of the interaction between fullerenes and the Li (or Li^+^) they may host? Detailed studies of combined systems are still highly desired, where Li or Li^+^ can be contained in fullerenes with various sizes.

This study is a theoretical investigation of endohedral fullerenes made with neutral or charged single atomic lithium (Li or Li^+^) and of fullerenes with various cage sizes, using the DFT self-consistent field molecular orbital (SCF-MO) method. We reveal the nature of interactions between Li (or Li^+^) and carbon cages based on interaction analysis.

## 2. Model and Computational Methods 

**Figure 1:**
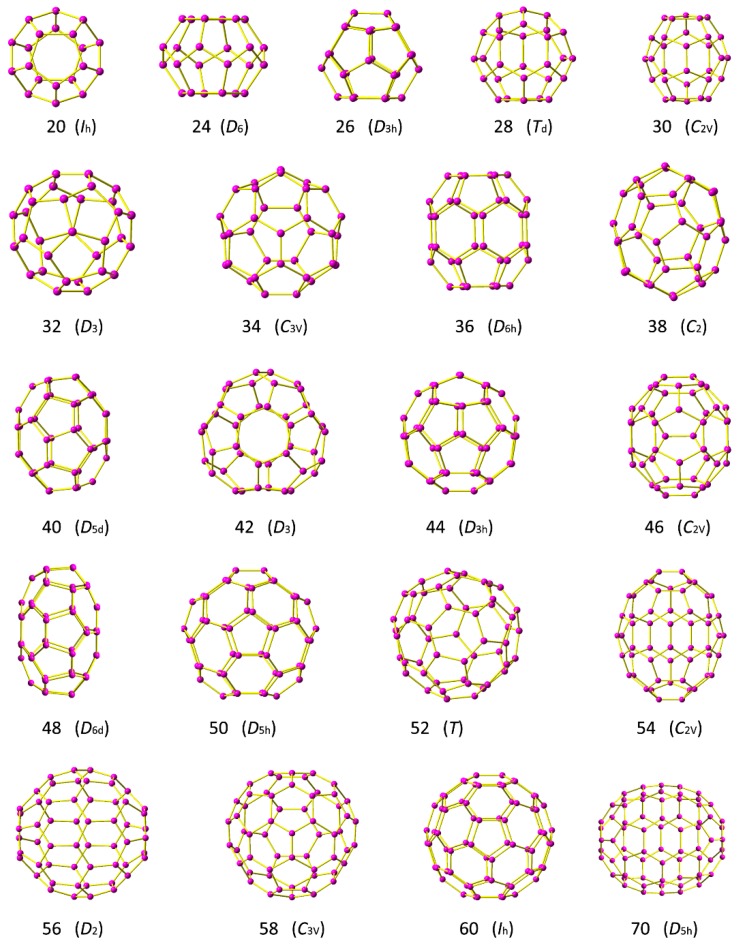
Structures of fullerene C*n* with *n* = 20–60, and 70.

In this study fullerene C*n* with *n* = 20–60 and 70 is systematically examined to host Li atom and Li cation. It is well-known that fullerenes have big families. Generally, each fullerene has many possible classic isomers. For instance, there are a total of 15 possible classical isomers for C_36_, while 1812 for C_60_ according to Euler’s theorem [29]. Notably, in this work one structure for each fullerene C*_n_* with *n* = 20–60 and 70 has been selected to capture the Li or Li^+^. As shown in the Figure 1, the structures with higher symmetries and stabilities generally have lower energy. Thus, these fullerene cages are selected to host Li and Li^+^. These fullerenes were also selected in previous studies [23,30,31]. 

The models of Li@C*n* and Li^+^@C*n* both at the central and off-central of the cage are considered. The models of the Li@C*n* and Li^+^@C*n* at the central are obtained firstly by DFT calculations. In order to get the off-central models, the annealing is performed by heating from 300 to 600 K and then cooling to 300 K with 5 cycles by using the molecular dynamics (MD) simulations based on the force filed method [32] by the Amsterdam Density Functional theory (*ADF*) package [33,34,35]. The calculations are conducted under a constant–temperature constant–volume condition with a temperature gradient of 0.025 K/step. Then the final version of the annealing MD is selected as an init structure submitted to DFT calculations. 

The calculations of geometry optimizations, energies and electronic properties of the endohedral fullerenes are performed by means of the DFT SCF-MO method throughout the work. The DFT functional M06-2X proposed by Zhao and Truhlar [36] is adopted due to its ability to effectively describe the weak interaction between the monomers. Besides M06-2X, different DFT methods such as PBEPBE and B3LYP have also been considered for Li@C_60_ and Li^+^@C_60_ as a comparison in this work. As shown in Figure S1 in Supplementary Material, the binding energy values of Li@C_60_ and Li^+^@C_60_ obtained by PBEPBE and B3LYP are both less negative than those by M06-2X, especially for the results by B3LYP. This agrees with previous DFT calculations. Grimme [37], et al. has studied the π–π stacked coronene with different DFT methods, and found that PBEPBE is not as preferable as M06-2X to describe the non-covalent interaction system. This study is also aware that B3LYP is not suitable to study the non-covalent interaction system from a quantitative view [37,38]. Besides, the dispersion-corrected M06-2X was also tested, and it was found that the value of *E*_b_ shares very similar values compared with the uncorrected ones. For instance, the differences between the corrected and uncorrected *E*_b_ are within 0.8 kcal/mol. 

All DFT calculations are performed at the double–ζ plus polarization basis sets 6–31 G (*d*, *p*) level implemented in *Gaussian 09* program (a comprehensive software package widely used in computational & quantum chemistry research, [39]). In the calculation of binding energy, deformation energy, and interaction energy, we adopted a higher integration accuracy than the default value in *Gaussian 09* program.

Due to the non-covalent bonding feature of the endohedral fullerenes in this work, we discuss the interaction using reduced density gradient (RDG [40]) with the help of multifunctional wave-function analyzer (Multiwfn [41]), a quantum chemical wave function analysis program and visual molecular dynamics (VMD [42]), a software for view and analyze molecular dynamics results. MD simulations were carried out by *ADF* package. 

## 3. Results

### 3.1. Energy and Stability

**Figure 2:**
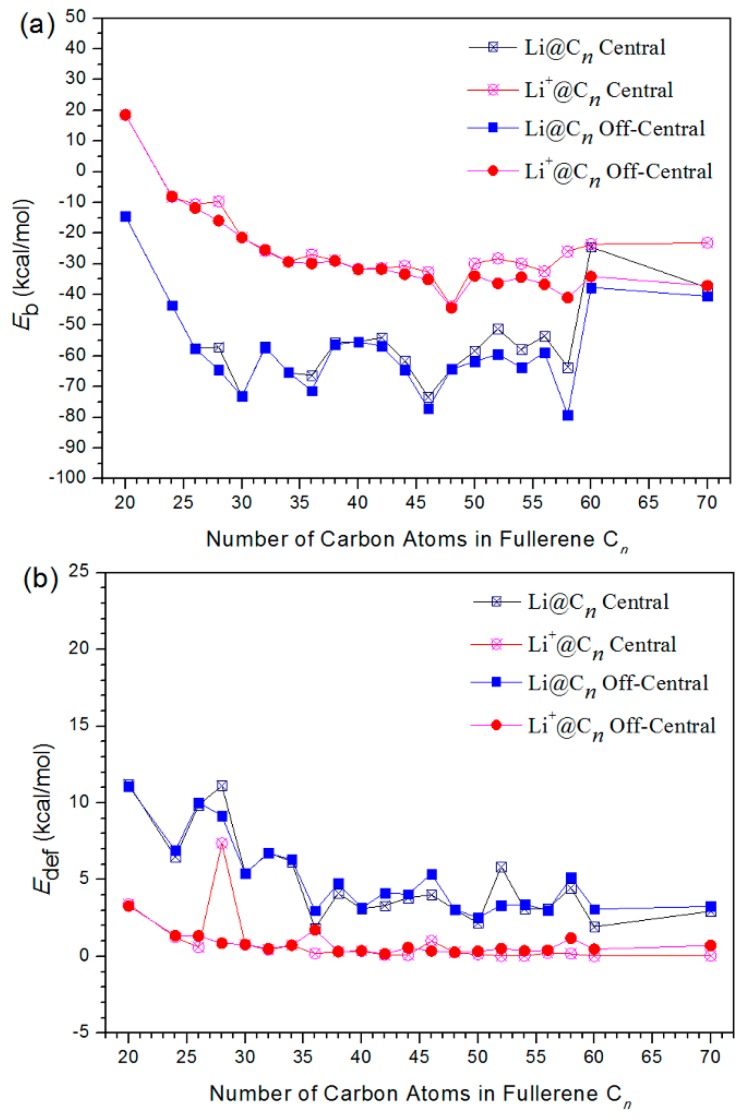
Binding energies (**a**) and deformation energies (**b**) of Li@C*n* and Li^+^@C*n*.

The obtained binding energies (*E*_b_) by DFT calculations are shown in Figure 2a, which represents the energies released in the process of forming the composite system by the two optimized monomers:

*E*_b_ = *E*_complex_ − *E*_cage-opt_ − *E*_Li_ (1)

where *E*_complex_, *E*_cage-opt_ and *E*_Li_ are the energies of the optimized geometries for the endohedral fullerenes complex, fullerene with minimum structure and atomic Li (or Li^+^), respectively.

Firstly, we focus on the neutral lithium atom encapsulated in a carbon cage (Li@C*n*). The values of *E*_b_ for Li@C*n* are all negative as shown in the figure, indicating that the encapsulation process of neutral lithium to fullerenes is energetically favorable. *E*_b_ are ranged from −14.6 to −73.4 kcal/mol with the Li atom is at the center of the cage, while *E*_b_ ranged from −14.5 to −79.3 kcal/mol when the Li atom was off-central from the cage. It is found that *E*_b_ is sensitive to the size of the cages. The values of *E*_b_ decrease very quickly (become more negative) when increasing the cage size up to *n* = 30. The larger the cage, the more the values for *E_b_* exhibits a fluctuation in form, with alternating increases and decreases. *E*_b_ of C*n* with *n* = 30, 36, 46 and 58 clearly present obvious more negative values than their neighbors whether the Li atom is central or not. This means that these carbon nanocages, compared to others, are even more energetically favorable to encapsulate the Li atom. Similar to the stabilities of fullerenes [43], 30, 36, 46, and 58 thus are the magic numbers to host the Li atom. In order to present the effect of Li or Li^+^ for the fullerene nanocages, we computed the formation energy of the empty cages (see Figure S2 of Supplementary Material). It is noticed that the magic numbers for the empty fullerenes are 28, 34, 48 and 58. This value indicates that Li or Li^+^ in the fullerene nanocages play an important role in stabilities of magic numbers for Li-based endohedral fullerenes. 

Turning to lithium cations hosted in carbon cages (Li^+^@C*n*), we found that *E*_b_ values for Li^+^@C*n* show a decreasing trend as the cages become larger. However, *E*_b_ of Li^+^@C*n* all exhibit less negative values compared to Li@C*n*. This means that the encapsulation of lithium cation is energetically less favorable than that of lithium atom. This fact is most obvious for Li^+^ hosted in small cages. For instances, *E*_b_ values for Li^+^@C_20_ are even positive (calculated to be about 18.5 kcal/mol), indicating an energetically unfavorable encapsulation process. 

In order to extend the studies, we also considered other alkaline metals, such as Na and K, hosted in the central of C_60_ cage. It is found that *E*_b_ of Na@C_60_ (Na^+^@C_60_) and K@C_60_ (K^+^@C_60_) are −32.9 (−25.8) and −56.9 (−29.2) kcal/mol, respectively. Thus the binding energies of K- and Na-based endohedral fullerenes are more negative than those of Li-based endohedral fullerenes. 

We also found that the off-central position of Li@C*n* and Li^+^@C*n* are energetically more favorable than the central position, according to the calculated *E*_b_. For very small cages, the difference of *E*_b_ for Li@C*n* and Li^+^@C*n* comparing central and off-central position, is so tiny as to be almost negligible. This can be attributed to the extremely limited space in small cages, where the off-central distances are very minute (<0.2 Å). However, according to the results of *E*_b_, Li@C_60_ and Li^+^@C_60_ have a clear tendency to depart from the central for larger cages. This can be attributed to the relatively larger space, where the off-central distances are greater (>1.0 Å). For instance, Li^+^ showed greater stability at off-center positions than at central positions, by 10.54 kcal/mol in Li^+^@C_60_, with an off-center distance of 1.399 Å. This agrees well with the finding that a lithium cation located at 1.34 Å off center in a C_60_ cage, according to prepared Li^+^@C_60_ in experiments [23] and previous DFT calculations [25,26]. Moreover, the off-central distance is 1.458 Å in Li@C_60_, greater than the 1.399 Å for Li^+^@C_60_. Encapsulated, Li^+^ and Li are located in the vicinity of a six-membered ring, which shows agreement with the pervious study [23]. It should be noticed that the *E_b_* of C_60_ changes quickly compared with those of C_58_. The sudden increase of *E*_b_ values (get less negative) for C_60_ is an interesting result, and the reason beyond such performance is, however, still unclear currently. 

We also noted that the encapsulation of Li@C*n* and Li^+^@C*n* would be concomitant with distortion in the carbon cages. To describe this distortion, the deformation energies (*E*_def_) can be defined as follows:

*E*_def_ = *E*_cage-def_ − *E*_cage-opt_ (2)

where *E*_cage-def_ represents the energy of empty cage in the optimized complex. The *E*_def_ value is obtained by measuring the energy difference between minimum energy structure and the energy of each empty cage in the optimized complex. The calculated results are shown in Figure 2b. The values of *E*_def_ are all positive, indicating that the deformation effect for all cages is endoergic and energetically unfavorable. This comes without much surprise, since that the carbon cages distort from their minimum energy structures during the formation of the Li@C*n* and Li^+^ @C*n*. *E*_def_ are in the range of 1.8–11.2 kcal/mol for Li@C*n*, and 0.03–7.4 kcal/mol for Li^+^@C*n*, respectively. *E*_def_ declined slowly in proportion to larger cage sizes. We are aware that *E*_def_ values for Li@C*n* all exhibit larger values than that of Li^+^@C*n*. This means that the distortion of the cage during encapsulation is more evident with the lithium atom than with the lithium cation. 

### 3.2. Interaction Analysis

Generally, the encapsulation of lithium (atom and cation) to carbon cages would include at least two processes. On the one hand, the cage would distort from the minimum energy structures as mentioned above, and on the other hand Li (or Li^+^) and deformed carbon cage would have interacted through several physical and chemical factors. These two processes usually are involved with each other. To reveal the energy released in the interaction process between the deformed components, the interaction energies (*E*_int_) are calculated by:

*E*_int_ = *E*_complex_ − *E*_cage-def_ − *E*_Li_ (3)

**Figure 3:**
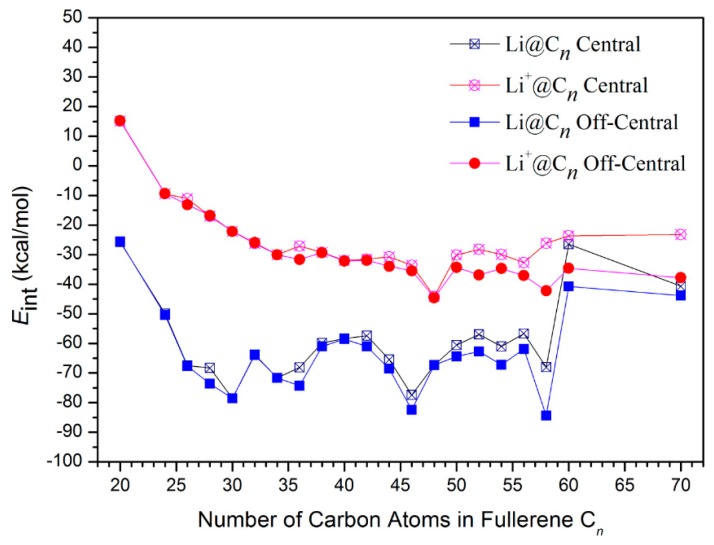
Interaction energies of Li@C*n* and Li^+^@C*n.*

The calculated results are shown in Figure 3. From the figures, it is clear that except for Li^+^@C_20_, the values of *E*_int_ for all Li@C*n* and Li^+^@C*n* complexes are all negative, indicating that the interaction process of both Li and Li^+^ with fullerenes is energetically favorable. However, *E*_int_ of Li^+^@C*n* all exhibit less negative values than that of Li@C*n*. This means that the interaction of lithium cation with carbon cages is energetically less favorable than that of lithium atom. The most notable point is that *E*_int_ shares exactly the same trend as that of *E*_b_. This means that *E*_b_ is mainly dependent on *E*_int_, rather than *E*_def_. Thus, there is no doubt that the interaction effect would dominate the whole encapsulation process of lithium to carbon cages.

**Figure 4:**
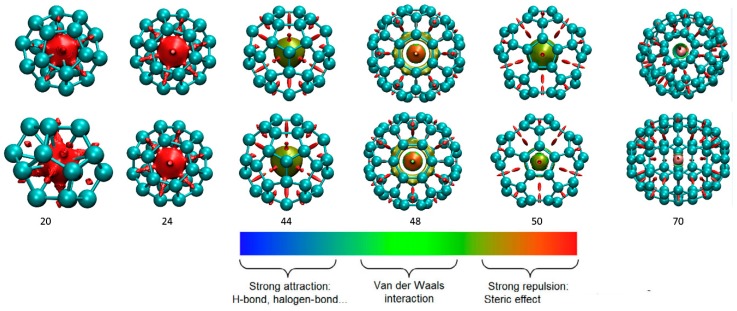
RDG of Li@C*n* (**up**) and Li^+^@C*n* (**down**).

In order to directly depict the physical image of the interaction between carbon cages and the hosted Li and Li^+^, here non-covalent interaction analysis is discussed based on the reduced density gradient (RDG). It is well known that RDG has become an effective tool to reveal the non-covalent interaction of various host-guest systems [44,45,46,47,48]. The visualizations of RDG for Li@C*n* and Li^+^@C*n* with *n* = 20, 24, 44, 48, 50 and 70 are shown in Figure 4. The interface color is clearly red for large regions in the visualizations for fullerenes C_20_ and C_24_, indicating stronger steric repulsion between Li or Li^+^ and carbon cages. The visualizations for fullerene C*n* = 44, 48 and 50 all exhibit a green interface color, meaning the Li and Li^+^ can be stabilized well by the van der Waals (VDW) interactions. Moreover, Li@C_50_ shows somewhat more surface color in green than does Li^+^@C_50_, indicating the stronger interaction for the Li atom than the Li cation in order to stabilize the complexes for these medium-sized cages. RDG of Li@C_70_ shows less green intensity due to the relatively large space within the cage, and this leads to the lower interaction with our findings for C_48_ and C_50_. These feathers revealed by RDG also agree well with the results based on *E*_int_ as discussed above. 

We also perform energy decomposition analysis (EDA) analysis in order to quantitatively describe the host-guest interaction for various Li-based endohedral fullerenes. For our purposes, *E*_int_ is decomposed to *E*_els_, *E*_ex_, *E*_orb_, and *E*_dis_ parts. Of these, *E*_els_ is the electrostatic part, describing the electrostatic interaction between the two monomers which contribute to the electronic density of the two isolated monomers directly superimposed; *E*_ex_ is the exchange repulsion term which designates the Pauli interaction, and shows a “repulsive” effect between the occupied orbit of one fragment and the occupied orbit of another; this repulsive effect results in an increase in the total energy, and in general, *E*_ex_ is a positive value; Finally, *E*_orb_ is the orbital part reflecting the interaction between the occupying orbit and other’s empty orbit, which will result in a lower energy. 

**Figure 5:**
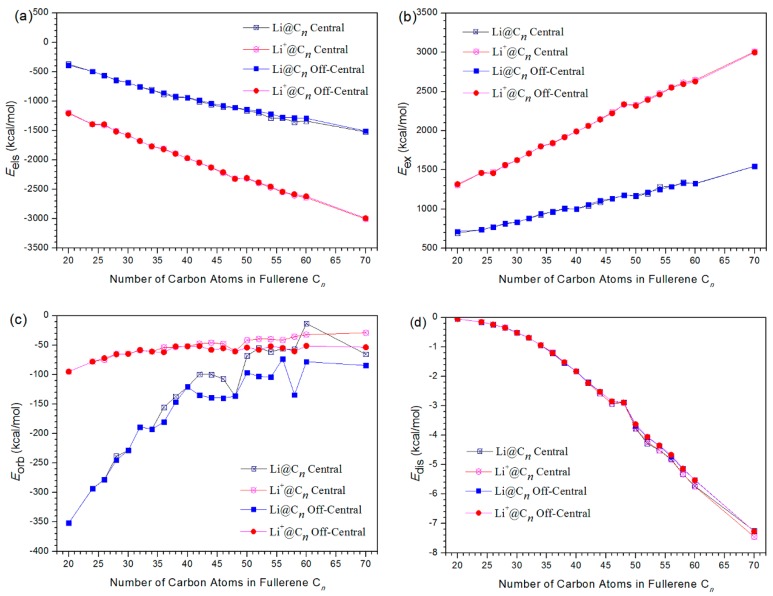
Electrostatic (**a**), exchange (**b**), orbital (**c**) and dispersion (**d**) interaction energies of Li@C*n* and Li^+^@C*n.*

We first consider electrostatic interaction. Usually, *E*_els_ is limited to the local charges between fragments, which will lead to a lower energy value. As a significant part in non-covalent interactions, electrostatic interactions can be attractive or repulsive. However, the electrostatic interaction in the host-gust nano systems is usually negative and therefore represents attraction [49,50,51]. It can be seen from Figure 5a that the values of *E*_els_ are all negative for both Li@C*n* and Li^+^@C*n*, indicating that electrostatic interaction is exothermic and energetically favorable to the complex. This is consistent with the results of charge transfers. It has been found that charge transfers occur in the formation of Li@C*n* and Li^+^@C*n*. and that the charge transfer ranges from 0.06 to 0.96 e_0_ for these Li-based endohedral fullerenes. It is well-known that the electrostatic interaction is an attractive force when two monomers respectively exhibit positive and negative charges due to the electron transfer from one to another [50]. The obtained *E*_els_ of Li@C*n* are ranged from −371.9 to −1522.9 kcal/mol for the central model (−389.6 to −1508.3 kcal/mol for the off-central model), and *E*_els_ of Li^+^@C*n* ranged from −1196.0 to −3003.2 kcal/mol for central model (−1209.4 to −2991.6 kcal/mol for the off-central model), respectively. Furthermore, it is evident that *E*_els_ for Li^+^@C*n* all exhibit more markedly negative values than do those for Li@C*n*. This means that the electrostatic interaction is a more important stabilizing factor for Li^+^@C*n* than it is for Li@C*n*. This can be attributed to the fact that Li cation itself has a higher charge than does the Li atom, and thus the electrostatic interaction is much stronger.

Then we turn to the exchange repulsion interaction term, which comes from the Pauli repulsion effect and is invariably positive. This is consistent with the physical nature that the exchange of electron as fermions presents a repulsion interaction. From the Figure 5b, it is found that the values of *E*_ex_ are all positive for both Li@C*n* and Li^+^@C*n*, this indicates that exchange repulsion interaction is energetically unfavorable to the complex. The obtained values for *E*_ex_ are in the range of 697.6–1547.2 kcal/mol for Li@C*n* (central model), and 1306.0–3009.3 kcal/mol for Li^+^@C*n* (central model), respectively. Notably, *E*_ex_ values for Li^+^@C*n* are all larger than those for Li@C*n*. This means that the exchange repulsion interaction is more likely to decrease the stability in Li^+^@C*n* than that of Li@C*n*. This can be confirmed from the fact Li^+^@C*n* is a close shell system with all electrons in pairs, but Li@C*n* is an open shell system with unpaired electrons.

It is also important to discuss the orbital interaction. The orbital interaction term is also known as induction term or polarization term. *E*_orb_ arises from the mix of occupied MOs and virtual MOs. If the combined Kohn–Sham wavefunction is used as initial guess for complex, then *E*_orb_ can be evaluated by subtracting the first SCF iteration energy from the last SCF iteration energy through the equation:

*E*_orb_ = *E*_SCF-Last_ − *E*_SCF-1st_

From Figure 5c, it is clear that all obtained *E*_orb_ are negative, whether for Li@C*n* or for Li^+^@C*n*. This means that orbital interaction, just like electrostatic interaction, is energetically favorable to the Li-based endohedral fullerenes. This result is also consistent with the interpretation nature of chemical bonds based on the energy decomposition analysis, in which the *E*_orb_ term is always attractive for the host-gust system since the total wave-function would be optimized during the SCF calculations. *E*_orb_ are ranged from −351.6 to −13.1 kcal/mol for Li@C*n* (central model), and ranged from −95.0 to −28.9 kcal/mol for Li^+^@C*n* (central model), respectively. It is revealed that except for C_60_, all exhibited *E*_orb_ values were more strongly negative for Li@C*n* than for Li^+^@Cn. This may result from the smaller electronic radius of lithium cation compared with the lithium atom when encountering the orbital interaction. This fact is the opposite of what we observed for *E*_els_, where Li^+^@C*n* values were more strongly negative. Moreover, the intensity of orbital interaction declined slowly as we examined larger cages, due to the less markedly negative *E*_orb_. 

Finally, we also considered dispersion. The calculated *E*_dis_ are shown in Figure 5d. As seen in the figure, *E*_dis_ ranged from −0.1 to −7.3 kcal/mol for Li@C*n* (central model), and −0.05 to −7.5 kcal/mol for Li^+^@C*n* (central model), respectively. The calculated *E*_dis_ are all negative values, and thus the dispersion interaction is also an attractive force. Furthermore, the intensity of dispersion interaction increased quickly as we examined increasingly large cages, due to the greater negative values for *E*_dis_. It is well-known that the dispersion interactions are among the important non-covalent interactions for many host-guest systems [44,45,46,47,48]. However, the values we calculated *E*_dis_ are within 8 kcal for both Li@C*n* and Li^+^@C*n* in this work. Compared with *E*_els_ and *E*_orb_ (which are also energetically favorable to the Li-based endohedral fullerenes in this wok), the values of *E*_dis_ are much smaller, and are possibly insignificant.

## 4. Conclusions

In this work we have studied the endohedral fullerenes made of neutral or charged single atomic lithium (Li or Li^+^) in fullerenes of various cage sizes by using first-principle DFT calculations. The nature of the interaction between Li@C*n* and Li^+^@C*n* is revealed based on interaction analysis of RDG calculations, along with EDA and charge transfer. 

The results of binding energies indicate that the encapsulation of lithium cation is energetically less favorable than that of lithium atom. The off-central position of Li and Li^+^ was energetically more favorable than at the centered position, and this fact agrees well with findings for prepared Li^+^@C_60_ in experiments, as well as with previous DFT calculations. We evaluated the encapsulation of lithium atoms and cations in carbon cages according to the separate effects of deformation and interaction, and we found that the latter of these effects dominates the whole encapsulation process of lithium to carbon cages.

RDG images directly depicted the physical interaction between carbon cages and the hosted Li or Li^+^. For EDA, the interaction effect was decomposed to *E*_els_, *E*_ex_, *E*_orb_ and *E*_dis_ parts in order to quantitatively describe the host-guest interaction for Li-based endohedral fullerenes. Electrostatic interaction is more important in the stabilization of the Li^+^@C*n* than that of Li@C*n*, due to the charged Li cations. The exchange repulsion interactions of Li^+^@C*n* were all greater than those of Li@C*n* since the latter exhibits an open-shell system with unpaired electrons. The orbital interaction of Li@C*n* almost always exhibits more strongly negative values than those of Li^+^@C*n*. Moreover, dispersion interaction is considered in this work, and it could be very insignificant to the interaction between Li or Li^+^ and carbon cages. 

## Supplementary Materials

The following are available online at https://www.mdpi.com/2079-4991/9/4/630/s1.
