# Supplementary material for: Interaction in Li@Fullerenes and Li+@Fullerenes: First Principle Insights to Li-Based Endohedral Fullerenes
Source: Nanomaterials (Basel). 2019 Apr 18;9(4):630. doi: 10.3390/nano9040630 (PMC6523142; doi:10.3390/nano9040630)
Supplement: Supplementary file 1 [file nanomaterials-09-00630-s001.pdf]

nanomaterials

Supplementary Material

Interaction in Li@Fullerenes and Li+@fullerenes: First Principle Insights to Li-based Endohedral Fullerenes

Hongcun Bai [1,] *, Hongfeng Gao [1], Wei Feng [1], Yaping Zhao [1] and Yuhua Wu [1]

[1] State Key Laboratory of High-efficiency Utilization of Coal and Green Chemical Engineering, College of Chemistry and Chemical Engineering, Ningxia University, Yinchuan 750021, Ningxia, China

* Correspondence: hongcunbai@gmail.com, hongcunbai@nxu.edu.cn; Tel.: +86-951-206-2328

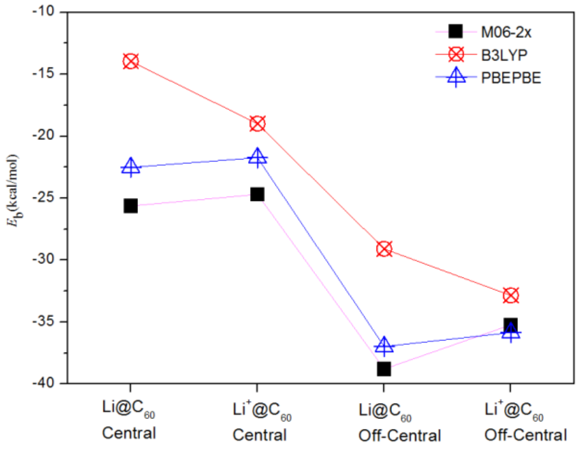

Figure S1. The binding energy of Li@C_{60} and Li^+@C_{60} obtained by PBEPBE, B3LYP and M06-2X methods

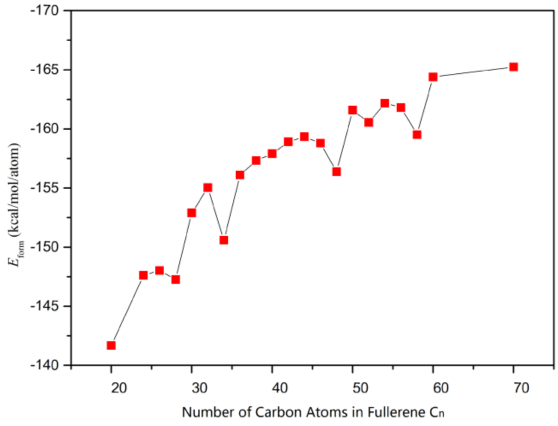

Figure S2. The formation energy of the empty cages (in kcal/mol/atom)
